# Strain-Specific Interactions of *Listeria monocytogenes* with the Autophagy System in Host Cells

**DOI:** 10.1371/journal.pone.0125856

**Published:** 2015-05-13

**Authors:** Marija Cemma, Grace Y. Lam, Martina Stöckli, Darren E. Higgins, John H. Brumell

**Affiliations:** 1 Cell Biology Program, Hospital for Sick Children, Toronto, Ontario M5G 1X8, Canada; 2 Department of Molecular Genetics, University of Toronto, Toronto, ON M5S 1A8, Canada; 3 Institute of Medical Science, University of Toronto, Toronto, ON M5S 1A8, Canada; 4 Department of Microbiology and Immunobiology, Harvard Medical School, Boston, Massachusetts 02115, United States of America; University of Illinois at Chicago College of Medicine, UNITED STATES

## Abstract

*Listeria monocytogenes* is an intracellular bacterial pathogen that can replicate in the cytosol of host cells. These bacteria undergo actin-based motility in the cytosol via expression of ActA, which recruits host actin-regulatory proteins to the bacterial surface. *L*. *monocytogenes* is thought to evade killing by autophagy using ActA-dependent mechanisms. ActA-independent mechanisms of autophagy evasion have also been proposed, but remain poorly understood. Here we examined autophagy of non-motile (Δ*actA*) mutants of *L*. *monocytogenes* strains 10403S and EGD-e, two commonly studied strains of this pathogen. The Δ*actA* mutants displayed accumulation of ubiquitinated proteins and p62/SQSTM1 on their surface. However, only strain EGD-e Δ*actA* displayed colocalization with the autophagy marker LC3 at 8 hours post infection. A bacteriostatic agent (chloramphenicol) was required for LC3 recruitment to 10403S Δ*actA*, suggesting that these bacteria produce a factor for autophagy evasion. Internalin K was proposed to block autophagy of *L*. *monocytogenes* in the cytosol of host cells. However, deletion of *inlK* in either the wild-type or Δ*actA* background of strain 10403S had no impact on autophagy evasion by bacteria, indicating it does not play an essential role in evading autophagy. Replication of Δ*actA* mutants of strain EGD-e and 10403S was comparable to their parent wild-type strain in macrophages. Thus, Δ*actA* mutants of *L*. *monocytogenes* can block killing by autophagy at a step downstream of protein ubiquitination and, in the case of strain EGD-e, downstream of LC3 recruitment to bacteria. Our findings highlight the strain-specific differences in the mechanisms that *L*. *monocytogenes* uses to evade killing by autophagy in host cells.

## Introduction

*Listeria monocytogenes* (*L*. *monocytogenes*) is the causative agent of listeriosis, a gastroenteritis that is self-limiting in healthy individuals but may become life-threatening for neonates/young children, pregnant women and immunocompromised individuals [1]. *L*. *monocytogenes* can infect a number of host cells, including macrophages [2]. Upon invasion, a population of bacteria is able to escape from the phagosome and colonize the nutrient-rich cytosol. Phagosomal escape is mediated by the bacterial pore forming toxin listeriolysin O (LLO) and two phospholipase C enzymes (PLCs). Upon entry into the cytosol, the bacteria use a cell surface protein, ActA, to drive actin-based motility in the host cytosol, and eventual cell-to-cell spread [2].

Clinical isolates of *L*. *monocytogenes* can be classified into 13 serovars based on gene content and virulence, and serotypes 1/2a, 1/2b and 4b are responsible for 95% of all human infections [3]. Many studies of *L*. *monocytogenes* utilize strains 10403S, a clinical isolate recovered from a skin lesion [4, 5], or EGD-e (ATCC BAA-679), which was originally isolated from rabbits [6, 7]. While these are both serotype 1/2a strains, genomic profiling reveals a high genetic diversity in this group of pathogens, suggesting they may have evolved strain-specific virulence factors [8]. A third strain commonly used for studies *L*. *monocytogenes* pathogenesis is strain EGD (NCTC7973). Genomic sequencing revealed that EGD has many genomic alterations and is actually more closely related to 10403S than EGD-e [9]. Importantly, the EGD strain harbors a lab-acquired mutation in the PrfA transcription factor (PfrA*) which results in constitutive expression of a number of virulence genes [9]. Therefore, studies using the EGD strain of *L*. *monocytogenes* must be carefully controlled and should not be compared directly to other strains with a wild type PrfA.

Previous studies by Webster and colleagues demonstrated that *L*. *monocytogenes* can be targets of macroautophagy (hereafter autophagy) while present in the cytosol of J774A.1 macrophage-like cells [10]. These authors utilized a non-motile ActA-deficient mutant (Δ*actA*, lacking the *actA* gene) of *L*. *monocytogenes* strain 10403S and infected cells for 3 hours (h). At this timepoint, the bacteriostatic antibiotic chloramphenicol (CM) was added to block protein synthesis and the infected cells were incubated a further 6 h before fixation and analysis by transmission electron microscopy. Webster and colleagues observed double-membrane autophagosomes containing *L*. *monocytogenes*, as well as intermediates of the autophagy process, such as isolation membranes enveloping bacteria. The number of viable intracellular bacteria decreased following CM addition, consistent with degradation of bacteria in autolysosomes. Their study was the first demonstration that cytosolic bacteria could be targets of autophagy. Subsequent studies using genetic approaches and the autophagy marker LC3 have firmly established the ability of the autophagy pathway to target bacteria [9, 11–13].

A recent study showed strain-specific differences in LC3 recruitment to two clones of Group A Streptococcus (GAS) [14]. Prior studies had established the role of autophagy as an innate defense system against the M6 clone of GAS, as LC3 conjugation to bacteria restricted their survival in non-phagocytic cells [12]. However, unlike the M6 clone, the M1T1 clone of GAS can efficiently replicate in the cytosol of infected cells by expressing a protease that degrades ubiquitin and ubiquitin-binding adaptors that target GAS to the autophagy pathway [14]. These studies suggest that different strains of bacterial pathogens, even those that are closely related at the taxonomic level, can have differential interactions with the autophagy system of host cells.

In this study we set out to examine potential strain-related differences in mechanisms of autophagy evasion in two of the most commonly used strains of *L*. *monocytogenes*, 10403S and EGD-e. Using the autophagy marker GFP-LC3 we confirmed that the Δ*actA* mutant of *L*. *monocytogenes* strain 10403S is targeted by autophagy within the cytosol in the presence of CM in RAW macrophages [15]. However, in the absence of CM we found that LC3 is not recruited, indicating other factors can mediate autophagy evasion. In fact, the Δ*actA* mutant of *L*. *monocytogenes* strain 10403S was previously shown to replicate normally within macrophages during the first 8 h post infection (p.i.) [16]. Conversely, we showed that wild-type bacteria were capable of evading autophagy in the presence of CM. This suggested that actin-based motility was sufficient to evade autophagy when protein synthesis was blocked. Therefore, *L*. *monocytogenes* strain 10403S appears to have both ActA-dependent and ActA–independent mechanisms to evade killing by autophagy during infection.

It is noteworthy that ActA protein expression is thought to occur only after bacteria have entered the cytosol [17]. Therefore, it has been speculated by Cossart and colleagues that ActA-independent mechanisms of autophagy evasion are important during the window of time after bacterial escape from the phagosome, but prior to ActA accumulation on the bacterial surface [18]. These authors suggested that bacterial expression of Internalin K (InlK) by *L*. *monocytogenes* strain EGD-e mediates autophagy evasion *in vivo*, but not *in vitro*, since InlK is only expressed in the former environment [18]. Furthermore, they proposed a mechanism whereby InlK recruitment of Major Vault Protein to the bacterial surface blocks protein ubiquitination events, and thereby prevents autophagy of bacteria in HeLa and RAW264.7 cell lines. It is noteworthy that in this study researchers alternated between using the EGD (NCTC7973) strain and the EGD-e strain. Interpreting their findings is problematic since subsequent genomic analysis revealed that the EGD strain harbors a PfrA* mutation which results in constitutive expression of a number of virulence genes [9], them and others have found that PrfA activation affects bacterial virulence [9, 19, 20]. Therefore, it remains unclear whether *L*. *monocytogenes* strains utilize InlK for autophagy evasion.

## Materials and Methods

### Reagents and Antibodies

Rabbit polyclonal antibodies against *L*. *monocytogenes* were a gift from Dr. Pascale Cossart (Institut Pasteur), mouse monoclonal antibodies against GFP were from Invitrogen (A-11120); antibodies against mouse p62-Ick ligand were from BD Biosciences (610832), rat polyclonal antibodies against LAMP1 were from Developmental Studies Hybridoma (1D4B) and antibodies against mono- and poly-ubiquitinated protein were from Biomol International (FK2; BML-PW8810-0500). All fluorescent secondary antibodies—goat anti rabbit 405, goat anti rat Cy3, goat anti mouse 568, goat anti mouse 488—were AlexaFluor conjugates from Molecular Probes (A31556; A10522, A11004; A11029; Invitrogen).

### Bacterial strains and tissue culture

Bacterial strains used in this study were as follows: wild-type *L*. *monocytogenes* 10403S [21], and isogenic mutants lacking LLO (Δ*hly*; DP-L2161) [22] and ActA (Δ*actA*; DP-L3078) [23]. 10403S Δ*inlK* and 10403S Δ*inlK*Δ*actA* strains were generated using standard protocols. EGD-e and isogenic EGD-e Δ*actA* strains were generously provided by Dr. Trinad Chakraborty [24]. RAW 264.7 macrophages were from American Type Culture Collection (TIB-71; Rockville, MD). RAW 264.7 cells were maintained in DMEM (SH30271.01; HyClone) supplemented with 10% FBS (090–510; Wisent) at 37°C in 5% CO_2_ without antibiotics. Macrophages were seeded at 1.25x10^5^ cells/well 48 h prior to infection.

### Bone marrow derived macrophage generation and culture conditions

All experimental protocols involving mice were approved by the Animal Care Committee of The Hospital for Sick Children. Mice were euthanized by cervical dislocation. The femur and tibia were removed, cleansed of muscle fibers and cut distally. The bone marrow was then removed via a 10 sec pulse of centrifugation at 2000 rpm. The resulting cells were centrifuged at 1500 rpm for 5 min, washed with growth media and plated on 10 cm tissue culture dishes. Media was replaced with fresh RPMI growth media (see below) supplemented with 30% L929 conditioned media every 3 days. 10^8^ bone marrow-derived macrophages (BMDM) were typically recovered after 7 days. Murine macrophages were maintained in RPMI-1640 medium (SH3002701; Wisent) supplemented with 10% FBS (090–510; Wisent), 5% sodium pyruvate (11360–070; Invitrogen), 5% antibiotics (15140122; Invitrogen), 5% non-essential amino acids (11140050; Invitrogen) and 0.5 μM ß-mercaptoethanol (21985023; Invitrogen). L929 conditioned medium was generated by growing L929 cells (CCL-1; ATCC) in 150-cm^2^ flasks at an initial density of 1x10^8^ cells per flask in growth media as described above. After 3 days, confluency was reached and the growth media was substituted with DMEM alone. After 7–10 days, culture supernatant was collected and centrifuged at 1,500 rpm for 5 min, aliquoted and stored at -20°C.

### Plasmids, transfections and siRNA silencing

Transfections were performed 24 h prior to infection. Transfection reagents FuGene 6 HD (04 709 691 001; Roche Applied Sciences) was used according to the manufacturers’ instructions. The GFP-LC3 construct was provided by Tamotsu Yoshimori (Osaka University, Japan) [25]. The GFP-LC3^G120A^ construct was provided by Walter Beron (Universidad Nacional de Cuyo).

### Bacterial infection conditions

Bacteria were grown in Brain-Heart Infusion (BHI) broth for 14–16 h at 30°C in a standing incubator. A 1:10 dilution of the culture was grown for 2 h at 37°C in a shaking incubator prior to infection. Both RAW 264.7 macrophages and BMDM were infected at a multiplicity of infection (MOI) of 10 as described [15].

### Replication Assay

Macrophages were plated at 1.25x10^5^ cells per well in 24-well tissue culture plates, 24 h prior to infection. All strains of *L*. *monocytogenes* were infected at a multiplicity of infection (MOI) of 10. After 30 min of invasion at 37°C, cells were washed three times with phosphate buffered saline (PBS) followed by the addition of DMEM. At 1 h post-infection, media was changed and growth media containing 50 μg/ml gentamicin was added. Cells were then lysed at indicated timepoints with 0.2% TritonX-100 in PBS. Serial dilutions of the lysates were plated on BHI-agar plates and incubated 14–16 h for subsequent quantification of colony forming units (CFUs).

### Immunofluorescence and microscopy

Immunostaining was conducted as previously described [26]. In brief, after infections, cells were fixed using 2.5% paraformaldehyde for 10 min at 37°C. Extracellular *L*. *monocytogenes* were detected by immunostaining prior to permeabilization. Cells were then permeabilized and blocked using 0.2% saponin with 10% normal goat serum for 14–16 h at 4°C. All colocalization quantifications were done using a Leica DMIRE2 epifluorescence microscope equipped with a 100X oil objective, 1.4 numerical aperture. 100 intracellular bacteria were examined in each experiment. Images are single confocal *z*-slices taken using a Zeiss Axiovert confocal microscope and LSM 510 software. Volocity software (Improvision) was used to analyze images. Images were imported into Adobe Photoshop and assembled in Adobe Illustrator.

### Statistical Analysis

Statistical analyses were conducted using GraphPad Prism v4.0a. In all figures, data is expressed as the mean ± standard deviation (SD) from three separate experiments. *P* values were calculated using two-tailed two-sample equal variance Student’s *t*-test. A p-value of less than 0.05 was considered statistically significant and is denoted by *. p < 0.01 is denoted by ** and p < 0.005 is denoted by ***. Data with multiple groups were analyzed using two-way ANOVA, then post hoc testing was performed with Bonferroni correction for multiple comparisons (GraphPad Prism v4.0a). Comparisons were considered significant if adjusted *P* < 0.05.

## Results

### LC3 recruitment to *L*. *monocytogenes* strains 10403S and EGD-e follows distinct kinetics

The Δ*actA* mutant of *L*. *monocytogenes* strain EGD-e was previously shown by Sasakawa and colleagues to recruit the ubiquitin-binding autophagy adaptor p62/SQSTM1 and become targets of autophagy, as judged by LC3 recruitment to bacteria [24]. These authors also observed a small, but statistically significant defect in the replication of these bacteria up to 4 h p.i. in MDCK cells. Since these authors did not use CM in their study, their findings are apparently at odds with previous observations showing that the Δ*actA* mutant of strain 10403S replicates normally in murine bone marrow-derived macrophages in the absence of CM [16]. We considered the possibility that strain specific factors impacting on bacterial interactions with the autophagy system in host cells may give rise to these apparently incongruent observations.

**Fig 1:**
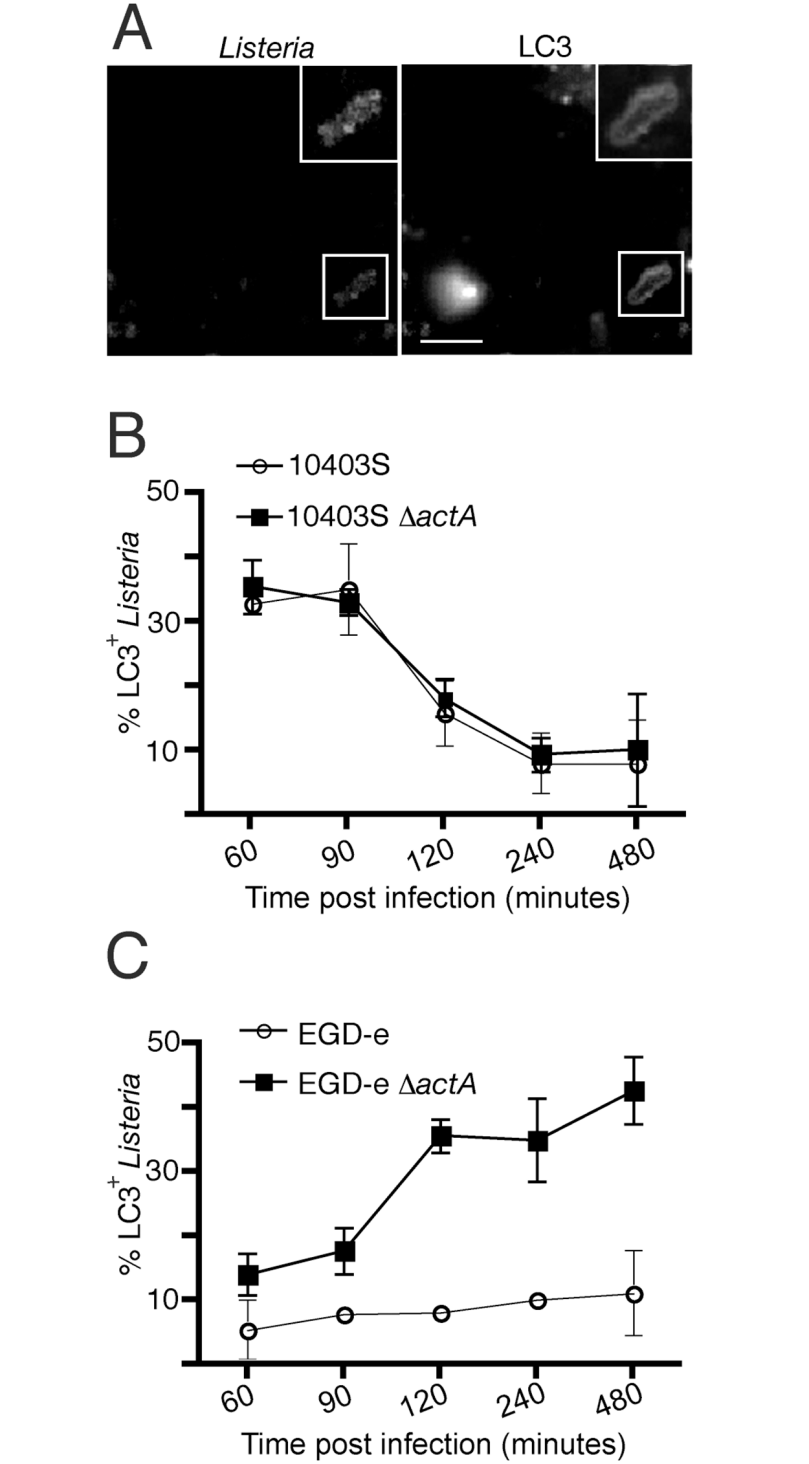
LC3 recruitment to *L*. *monocytogenes* strains 10403S and EGD-e follows distinct kinetics. (A) Confocal images of RAW 264.7 macrophages transfected with GFP-LC3 and infected for 1 h with wild-type 10403S *L*. *monocytogenes*. Scale bar: 5 μm. (B) Quantification of LC3 colocalization with intracellular wild-type 10403S or isogenic Δ*actA* mutant bacteria over time in RAW 264.7 macrophages. (C) Quantification of LC3 colocalization with intracellular wild-type EGD-e or isogenic Δ*actA* mutant bacteria over time in RAW 264.7 macrophages.

We infected RAW 264.7 macrophages expressing GFP-LC3 with wild-type strains of 10403S or EGD-e *L*. *monocytogenes*. Using this approach, we observed LC3 recruitment to strain 10403S that peaked at 1 h p.i., consistent with previous studies [15, 27, 28]. In contrast, wild-type strain EGD-e displayed negligible recruitment of LC3 throughout infection, as previously reported in MDCK cells [24, 29] (Fig 1A–1C). Our findings confirm the apparent differences of LC3 recruitment to different *L*. *monocytogenes* strains reported in the literature and suggest that wild-type strain EGD-e is capable of preventing LC3 recruitment to bacteria at 1 h p.i.. Importantly, both wild-type strains displayed little colocalization with LC3 at later stages of infection, when bacteria are replicating rapidly in the cytosol. Therefore, both wild-type strains of *L*. *monocytogenes* have effective mechanisms to evade autophagy in the cytosol.

Next, we examined LC3 recruitment to Δ*actA* mutants of the two *L*. *monocytogenes* strains. Sasakawa and colleagues observed that the Δ*actA* mutant of EGD-e showed increased LC3 colocalization at 2 and 4h p.i. in MDCK cells [24] while a study conducted by Birmingham *et al*. showed that the Δ*actA* mutant of 10403S showed no differences in LC3 colocalization compared to parent wild-type bacteria in RAW 264.7 macrophages [15, 27]. Here, we confirm all of these previous findings in our laboratory using RAW 264.7 macrophages as we observed the Δ*actA* mutant of EGD-e recruited LC3 at later stages of infection while the Δ*actA* mutant of 10403S did not (Fig 1B and 1C). Importantly, our studies were performed ‘head-to-head’ under identical experimental conditions, suggesting that strain-specific factors underly the differences in LC3 recruitment to bacteria, and not differences in cell-type or experimental protocols in prior studies. We conclude that LC3 recruitment to *L*. *monocytogenes* strains 10403S and EGD-e follows distinct kinetics.

### Lipidation of LC3 is required for its recruitment to the Δ*actA* mutant of *L*. *monocytogenes* strain EGD-e

**Fig 2:**
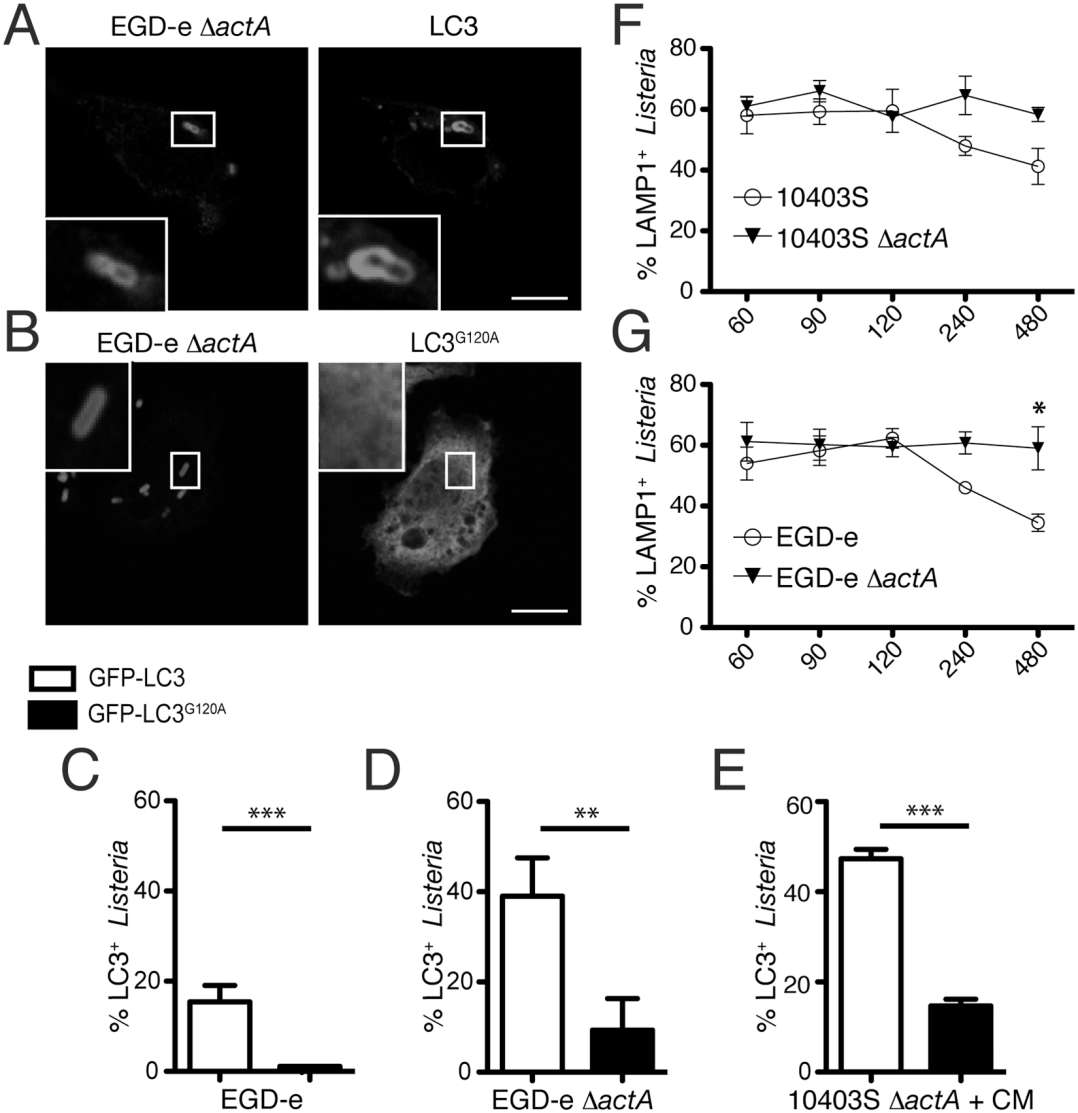
Lipidation of LC3 is required for its recruitment to the Δ*actA* mutant of *L*. *monocytogenes* strain EGD-e. (A, B) Confocal images of RAW 264.7 macrophages transfected with GFP-LC3 (A) or GFP-LC3^G120A^ (B) and infected for 4 h with EGD-e Δ*actA L*. *monocytogenes*. Scale bar: 5 μm. (C,D) Quantification of LC3 or GFP-LC3^G120A^ colocalization with intracellular wild-type EGD-e (C) or EGD-e Δ*actA* (D) bacteria at 4 h p.i. in RAW 264.7 macrophages. (E) Quantification of LC3 or GFP-LC3^G120A^ colocalization with intracellular 10403S Δ*actA* bacteria infected for 3 h, followed by 5 h CM treatment (+CM) in RAW 264.7 macrophages. (F) Quantification of LAMP1 colocalization with intracellular wild-type 10403S or isogenic Δ*actA* mutant bacteria from 1 to 8 h p.i. in RAW 264.7 macrophages. (G) Quantification of LAMP1 colocalization with intracellular wild-type EGD-e or isogenic Δ*actA* mutant bacteria from 1 to 8 h p.i. in RAW 264.7 macrophages.

When LC3 is recruited to the autophagic membrane it undergoes a cleavage and covalent conjugation to phosphoethanolamine on its C-terminal Glycine residue. Thus, LC3 can be found in two forms, a cytosolic LC3-I form and membrane-bound LC3-II form. To distinguish between these two possibilities, we have used an LC3 mutant, GFP-LC3G^120A^, which is a non-conjugatable mutant of LC3. RAW 264.7 macrophages were transfected with either GFP-LC3 or GFP-LC3G^120A^ and infected with wild-type EGD-e or Δ*actA* EGD-e for 4 h. As a positive control, we infected cells with the Δ*actA* mutant of strain 10403S for 8h with CM treatment at 3h p.i. (Fig 2E). In all cases there was significantly fewer LC3^+^ *L*. *monocytogenes* when cells were transfected with the non-conjugatable version, LC3^G120A^, when compared to the wild-type LC3 (Fig 2A–2E). Therefore, optimal LC3 recruitment to the Δ*actA* mutant of EGD-e strain requires its lipidation. This observation suggested that LC3 was associated with membranes surrounding bacteria. To test this hypothesis we examined LAMP1, a late endosome marker, in infected cells. We observed that at the time of LC3 recruitment, (2–8 h p.i.) ≈ 60% of the Δ*actA* mutants of EGD-e strain colocalized with LAMP1 (Fig 2F and 2G). Together, these data suggest that LC3^+^ Δ*actA* mutants of EGD-e were present in membrane-bound compartments.

### ActA prevents association of ubiquitinated proteins and p62/SQSTM1 with *L*. *monocytogenes* in a strain-independent manner

**Fig 3:**
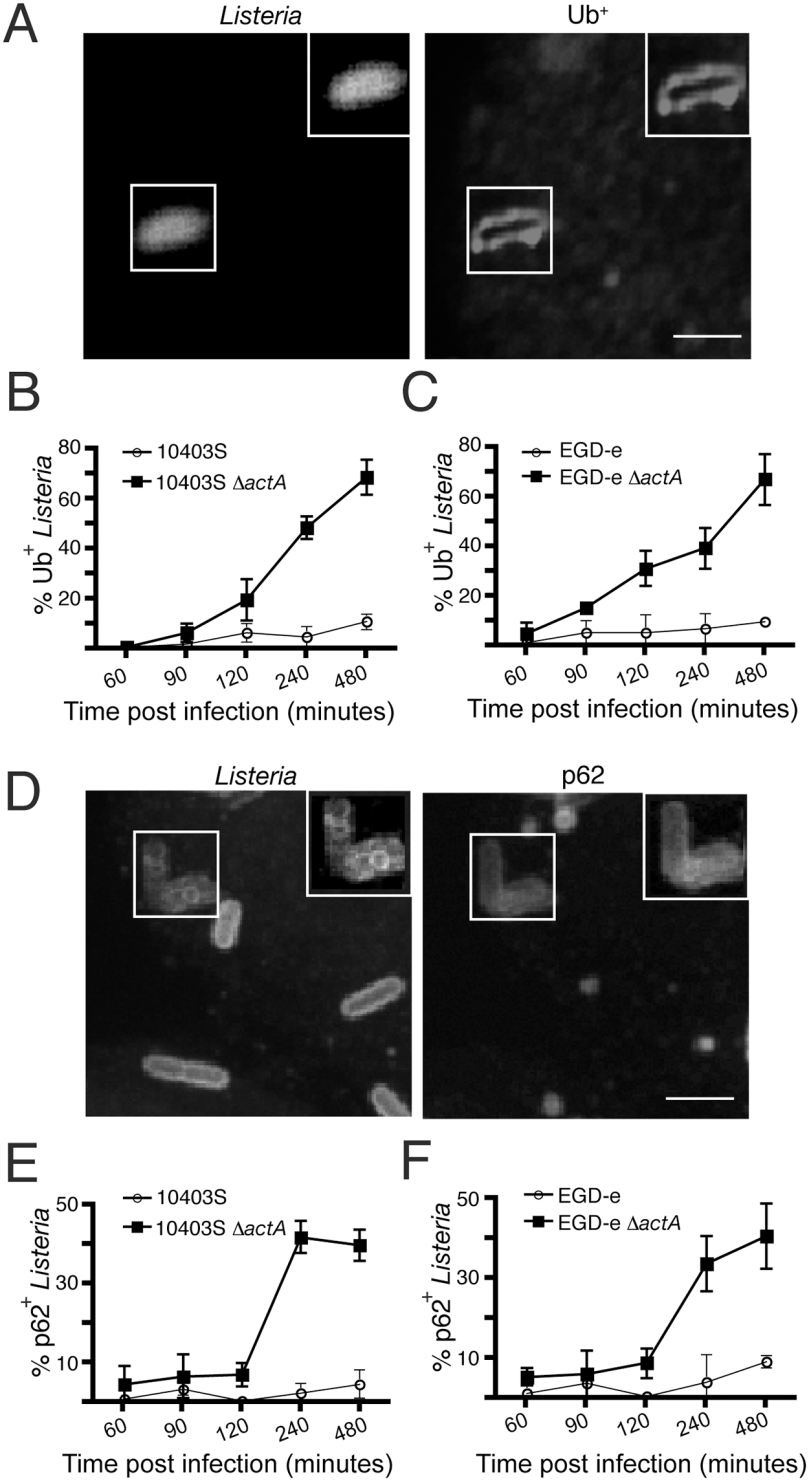
ActA prevents association of ubiquitinated proteins and p62/SQSTM1 with *L*. *monocytogenes* in a strain-independent manner. (A) Confocal images of RAW 264.7 macrophages infected for 1 h with wild-type 10403S *L*. *monocytogenes*, stained for Ub. (B) Quantification of Ub colocalization with intracellular wild-type 10403S or 10403S Δ*actA* bacteria over time. (C) Quantification of Ub colocalization with intracellular wild-type EGD-e or EGD-e Δ*actA* bacteria over time. (D) Confocal images of RAW 264.7 macrophages infected for 1 h with wild-type EGD-e *L*. *monocytogenes*, stained for p62/SQSTM1. (E) Quantification of p62/SQSTM1 colocalization with intracellular wild-type 10403S or 10403S Δ*actA* bacteria over time. (F) Quantification of p62/SQSTMI colocalization with intracellular wild-type EGD-e or EGD-e Δ*actA* bacteria over time. Scale bar: 5 μm.

The accumulation of ubiquitinated (Ub^+^) proteins is known to be a signal for selective autophagy of cytoplasmic contents, including bacteria [30]. Therefore, we examined the co-localization with Ub^+^ proteins with both strains of *L*. *monocytogenes*. The two wild-type strains displayed little colocalization with of Ub^+^ proteins throughout infection (Fig 3B and 3C). However, the Δ*actA* mutants of both strains colocalized with Ub^+^ proteins, beginning at 90 min p.i. and peaking at 8 h p.i., when ≈ 70% of bacteria displayed strong colocalization with Ub^+^ proteins (Fig 3A–3C). This is consistent with previous studies of the Δ*actA* mutant of *L*. *monocytogenes* strain LO28 [31]. We conclude that *L*. *monocytogenes* can block the association of Ub^+^ proteins with the bacterial surface via ActA expression in a strain-independent manner.

Selective autophagy of cytoplasmic contents involves association of ubiquitinated proteins with ubiquitin-binding autophagy adaptors such as p62/SQSTMI [30]. Therefore, we examined p62/SQSTM1 recruitment to both *L*. *monocytogenes* strains. We observed negligible recruitment to either wild-type strain during infection (Fig 3D–3F). In contrast, p62/SQSTM1 was recruited to Δ*actA* mutants of both strains, with recruitment kinetics similar to that of Ub^+^ protein association. Sasakawa and colleagues showed that LC3 recruitment to the Δ*actA* mutant of EGD-e requires p62/SQSTM1 expression [24]. Since the Δ*actA* mutant of strain 10403S displays recruitment of Ub^+^ proteins and p62/SQSTM1 but not LC3, it suggests that 10403S can actively block autophagy at a step downstream of adaptor recruitment.

### The Δ*actA* mutant of *L*. *monocytogenes* evades autophagy in a protein synthesis-dependent manner

**Fig 4:**
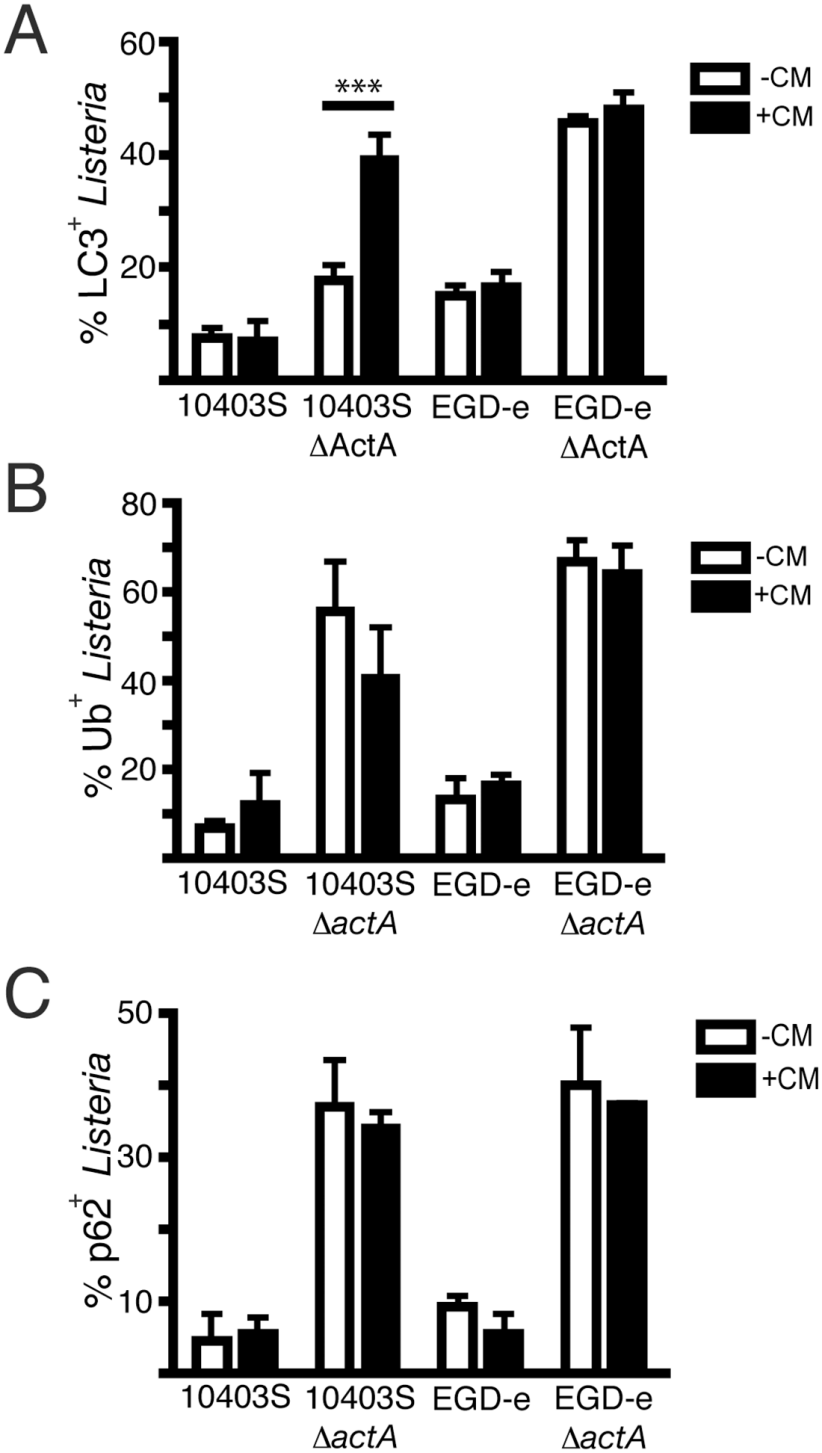
The Δ*actA* mutant of *L*. *monocytogenes* evades autophagy in a protein synthesis-dependent manner. (A) Quantification of the percentage of *L*. *monocytogenes* that are LC3^+^ in RAW 264.7 macrophages infected with either 10403S, 10403S Δ*actA*, EGD-e, EGD-e Δ*actA* bacteria for: 8 h (-CM) or 3 h, followed by 5 h CM treatment (+CM). *** P value is < 0.0001 (two-way ANOVA with Bonferroni correction) (B) Quantification of the percentage of *L*. *monocytogenes* that are Ub^+^ in RAW 264.7 macrophages infected with either 10403S, 10403S Δ*actA*, EGD-e, EGD-e Δ*actA* bacteria for: 8 h (-CM) or 3 h, followed by 5 h CM treatment (+CM). (C) Quantification of the percentage of *L*. *monocytogenes* that are p62/SQSTMI^+^ in RAW 264.7 macrophages infected with either 10403S, 10403S Δ*actA*, EGD-e, EGD-e Δ*actA* bacteria for: 8 h (-CM) or 3 h, followed by 5 h CM treatment (+CM).

Next, we focused on how the Δ*actA* mutant of 10403S strain evades autophagy in the cytosol. When chloramphenicol (CM), a bacterial protein synthesis inhibitor, was added to the RAW 264.7 macrophages at 3 h p.i., we found a significant increase in LC3 colocalization with the 10403S Δ*actA* but not the 10403S wild-type strain (Fig 4A), consistent with previous findings [10, 15]. This observation suggests that in the absence of ActA, the 10403S Δ*actA* strain produces an additional bacterial factor which aides in the evasion of autophagy. In contrast, CM treatment had no effect on LC3 recruitment to the Δ*actA* mutant of strain EGD-e (Fig 4A). Association of ubiquitinated proteins and p62/SQSTMI with bacteria was not significantly different following CM treatment for any of the strains tested (Fig 4B and 4C).

### InlK is not required for autophagy evasion by the Δ*actA* mutant of *L*. *monocytogenes* strain 10403S

**Fig 5:**
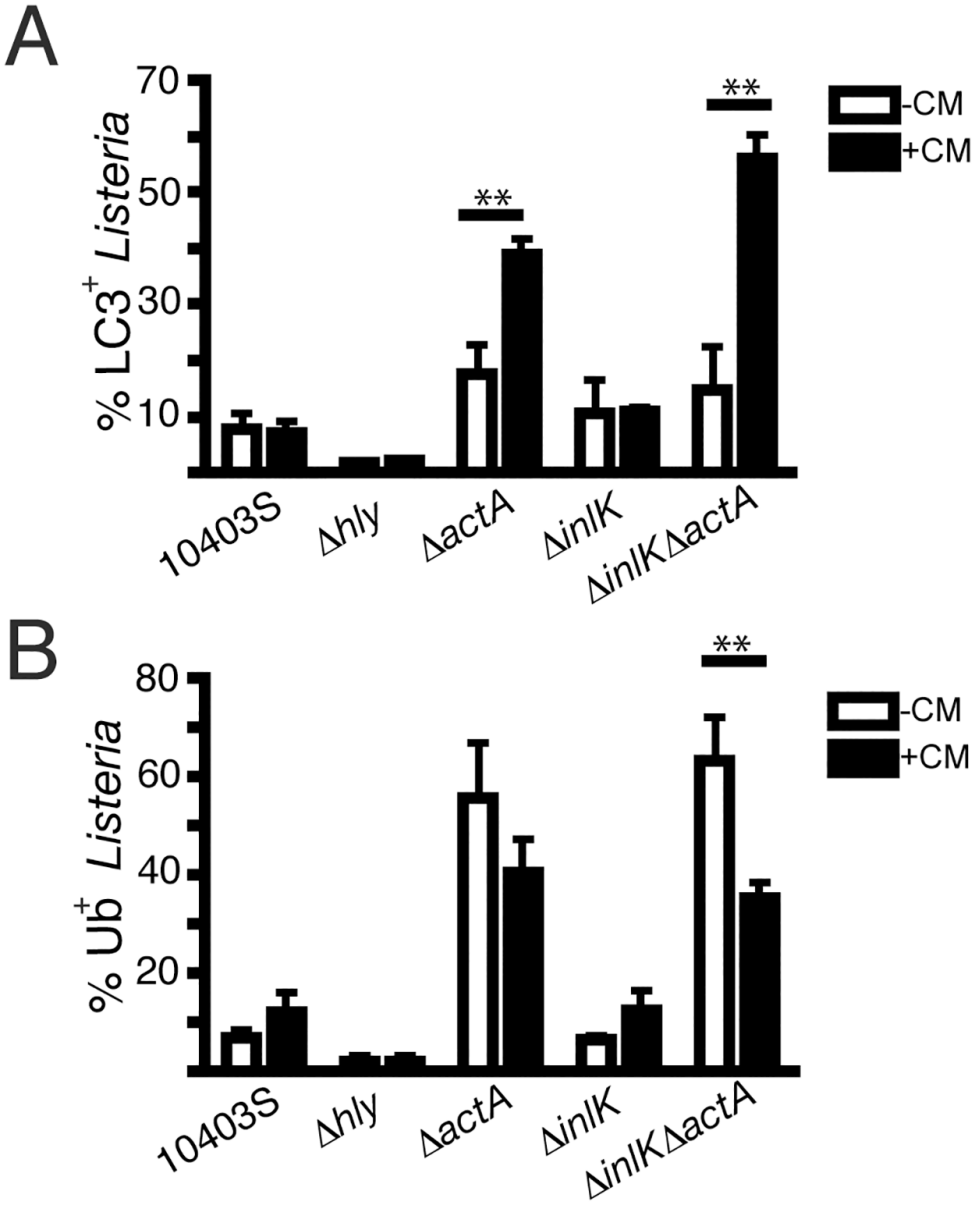
InlK does not prevent association of ubiquitinated proteins with *L*. *monocytogenes* strain 10403S. (A) Quantification of the percentage of *L*. *monocytogenes* that are LC3^+^ in RAW 264.7 macrophages infected with *L*. *monocytogenes* with the 10403S background: wildtype (10403S), LLO deficient (Δ*hly*), ActA deficient (Δ*actA*), InlK deficient (Δ*inlK*) and InlK and ActA deficient (Δ*inlK*Δ*actA*): 8 h (-CM) or 3 h, followed by 5 h CM treatment (+CM). **P value is < 0.001 (two-way ANOVA with Bonferroni correction). (B) Quantification of the percentage of *L*. *monocytogenes* that are LC3^+^ in RAW 264.7 macrophages infected with *L*. *monocytogenes* with the 10403S background: wildtype (10403S), LLO deficient (Δ*hly*), ActA deficient (Δ*actA*), InlK deficient (Δ*inlK*) and InlK and ActA deficient (Δ*inlK*Δ*actA*): 8 h (-CM) or 3 h, followed by 5 h CM treatment (+CM). ***P value is < 0.001 (two-way ANOVA with Bonferroni correction).

Cossart and colleagues recently suggested that the bacterial virulence factor InlK may contribute to the evasion of autophagy by *L*. *monocytogenes* EGD-e [18]. Thus, we tested a possible role for InlK in autophagy evasion by strain 10403S. 10403S mutants deficient in either InlK (Δ*inlK*) or InlK and ActA (Δ*inlK*Δ*actA*) did not exhibit any differences in LC3 colocalization compared to wild-type bacteria (Fig 5A). We conclude that InlK does not play an essential role in autophagy evasion by *L*. *monocytogenes* strain 10403S in the presence or absence of ActA.

Next, we examined the role of InlK in autophagy evasion by strain 10403S when bacterial protein synthesis was inhibited with CM. The Δ*inlK* mutant did not exhibit an increase in LC3 colocalization over the untreated control (Fig 5A). However, the Δ*inlK*Δ*actA* strain exhibited a significant increase in LC3 colocalization over its untreated control. This observation suggests that ActA, and not InlK, is critical for the evasion of LC3 recruitment in the presence of CM. It is noteworthy that in the presence of CM there was an increase in LC3 recruitment to the Δ*inlK*Δ*actA* mutant compared to the Δ*actA* mutant of strain 10403S *L*. *monocytogenes* in the presence of CM (35% LC3^+^ Δ*actA* vs. 55% LC3^+^ Δ*inlK*Δ*actA*). This suggests that InlK may play a redundant role in autophagy evasion by the Δ*actA* mutant of strain 10403S that only becomes apparent after CM treatment.

### InlK does not prevent association of ubiquitinated proteins with *L*. *monocytogenes* strain 10403S

Cossart and colleagues suggested that InlK promotes autophagy evasion by strain EGD-e by recruitment of the Major Vault Protein (MVP) to the bacterial surface, thereby ‘shielding’ bacteria from protein ubiquitination and ubiquitin-binding autophagy adaptor recruitment [18]. Therefore, we examined Ub^+^ protein association with the 10403S strain mutants (Fig 5B). The Δ*inlK* mutant did not exhibit a change in Ub^+^ protein association with bacteria either with or without CM treatment when compared to wild-type bacteria. Remarkably, the Δ*inlK*Δ*actA* double mutant displayed less colocalization with Ub^+^ protein in the presence of CM compared to the absence of CM. This was surprising since these conditions lead to increased LC3 recruitment to bacteria (Fig 5A). Furthermore, we did not observe recruitment of MVP to the surface of either wild-type or Δ*actA L*. *monocytogenes* 10403S (data not shown). These findings suggest that InlK may contribute to autophagy evasion in a redundant manner with ActA and other bacterial factors, but that its mechanism of action does not involve blocking Ub^+^ protein association with bacteria.

### Δ*actA* mutants of *L*. *monocytogenes* strains 10403S and EGD-e replicate rapidly in macrophages

**Fig 6:**
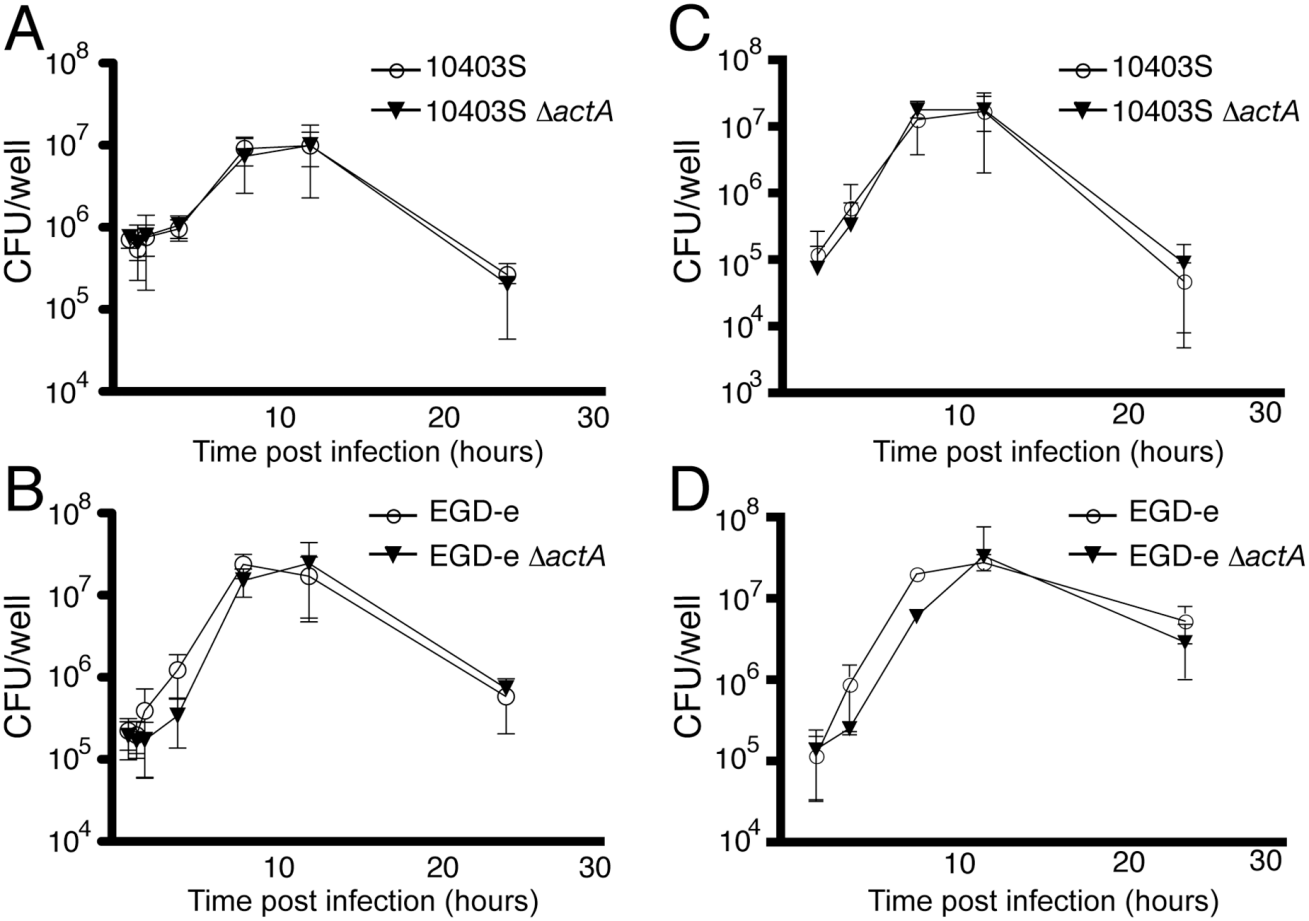
Δ*actA* mutants of *L*. *monocytogenes* strains 10403S and EGD-e can replicate rapidly. (A) RAW 264.7 macrophages were infected with 10403S or 10403S Δ*actA L*. *monocytogenes* in a replication assay. The number of bacteria per infected well (CFU/well) were quantified at 1, 1.5, 2, 4, 8, 12, and 24h p.i.. (B) RAW 264.7 macrophages were infected with EGD-e or EGD-e Δ*actA L*. *monocytogenes* in a replication assay. The number of bacteria per infected well (CFU/well) were quantified at 1, 1.5, 2, 4, 8, 12, and 24h p.i.. (C) BMDM from C57/B6 mice were infected with 10403S or 10403S Δ*actA L*. *monocytogenes* in a replication assay. The number of bacteria per infected well (CFU/well) were quantified at 2, 4, 8, 12, and 24h p.i.. (D) BMDM from C57/B6 mice were infected with EGD-e or EGD-e Δ*actA L*. *monocytogenes* in a replication assay. The number of bacteria per infected well (CFU/well) were quantified at 2, 4, 8, 12, and 24h p.i..

Bacterial replication assays were performed to quantify the numbers of bacteria in infected RAW 264.7 macrophages. The Δ*actA* mutants of *L*. *monocytogenes* strains 10403S and EGD-e replicated normally in RAW 264.7 macrophages compared to parent wild-type bacteria (Fig 6A and 6B), consistent with previous findings [16]. In contrast, the Δ*actA* mutant of strain EGD-e displayed a slight replication defect compared to parent wild-type bacteria during the first 4 h p.i.. These findings are consistent with previous studies by Sasakawa and colleagues who studied the early stages of infection (up to 4 h p.i.) in MDCK cells [24]. However, we found that this replication defect was minor, and was absent by 8 h p.i., when the Δ*actA* mutant achieved similar intracellular bacterial numbers compared to parent wild-type bacteria (Fig 6B). Similar results were observed in bone marrow-derived macrophages (BMDMs)(Fig 6C and 6D). Therefore, despite marked colocalization of LC3 with the Δ*actA* mutant of EDG-e, these bacteria are not cleared from the cytosol, but rather are still capable of rapid replication in the cytosol of host cells. These findings indicate the existence of other mechanisms that prevent bacterial delivery to the lysosome even after LC3 targeting to the Δ*actA* mutant of *L*. *monocytogenes* EGD-e.

## Discussion

Previous studies by Webster and colleagues established that, under certain laboratory conditions, *L*. *monocytogenes* can be targeted by autophagy within the cytosol [10]. This was a seminal study that helped to establish an important role for autophagy in innate immunity. Since their study, it has been shown that *L*. *monocytogenes* has multiple mechanisms to evade autophagy during infection. ActA expression by bacteria is likely to mediate autophagy evasion by several mechanisms. First, actin polymerization on the bacterial surface has been suggested to provide a protective ‘shield’ for bacteria [24]. Second, actin-based motility allows bacteria to move rapidly in the cytosol, possibly allowing bacteria to escape capture in autophagosomes. Third, ActA expression by *L*. *monocytogenes* prevents the accumulation of ubiquitinated proteins on the bacterial surface [31]. Fourth, bacterial sequestration of Arp2/3, which is required for autophagy in yeast [32], may impair actin polymerization events required for autophagic capture of bacteria. Thus, we conclude that ActA, a major virulence factor found in all pathogenic *L*. *monocytogenes*, plays a key role in autophagy evasion, in addition to its established role in cell-to-cell spread. ActA expression has been shown to be upregulated only after bacteria have entered the cytosol [17]. Therefore, it has been speculated that ActA-independent mechanisms of autophagy evasion are important during the window of time after phagosome escape but prior to ActA accumulation on the bacterial surface [18].

Our study indicates that ActA-independent mechanisms of autophagy evasion are also multifactorial, and strain-dependent. We found striking evidence that the 10403S strain of *L*. *monocytogenes* interacts differently with the host autophagy pathway compared to the EGD-e strain. We propose that after strain 10403S has disrupted the phagosome and gained access to the cytosol, it evades autophagy via several pathways—one of which is dependent on ActA and at least two that are ActA-independent. In the absence of ActA expression, this second pathway can actively evade autophagy, despite accumulation of Ub^+^ protein and p62/SQSTM1 on the bacterial surface. The Δ*actA* mutant of EGD-e *L*. *monocytogenes* recruits LC3 efficiently after access to the cytosol. Despite this targeting, replication of Δ*actA* mutants of strain EGD-e and 10403S was comparable to their parent wild-type strain in murine macrophages. Thus, Δ*actA* mutants of *L*. *monocytogenes* can block killing by autophagy at a step downstream of protein ubiquitination and LC3 recruitment to bacteria.

In our study, InlK was found to play a minor role in autophagy evasion by the Δ*actA* mutant of strain 10403S, but only in the presence of CM. A previous study suggested that InlK inhibited antimicrobial autophagy via the recruitment of the major vault protein complex [18]. In that study, InlK was found to promote *L*. *monocytogenes* virulence in a mouse model of systemic disease using the EGD-e strain [18]. However, the *in vivo* role for InlK was not linked the autophagy pathway, as only wild type mice were used in the study [18]. Overexpression of InlK was found to promote growth of *L. monocytogenes in vitro [18]*. However, the role of InlK in evading autophagy was not adequately assessed since none of the conventional assays for autophagy quantification of LC3-positive bacteria, quantification of vacuolar bacteria or electron microscopy were performed. Furthermore, the knockout of InlK was not examined since it is not expressed by bacteria *in vitro*. While InlK overexpression enhanced bacterial replication of the Δ*actA* mutant, this experiment was performed with the EGD strain, a genetically distinct *L*. *monocytogenes* strain from EGD-e [9], and the replication difference was not directly linked to autophagy [18]. Therefore, we conclude that InlK has a minor, if any, role in the autophagy evasion in the 10403S strain.

Our study highlights the multiple mechanisms used by *L*. *monocytogenes* to evade killing by autophagy, as well as the fact that some of these mechanisms are strain-specific. Our findings point to evolutionary pressure on the pathogens to modulate autophagy. Future genomic analysis of clinically-relevant strains of *L*. *monocytogenes* are likely to yield other virulence factors that mediate ActA-independent autophagy evasion. However, our study indicates that multiple gene knockouts (e.g. Δ*actA*Δ*geneX*Δ*geneY*) will be required to establish their role in autophagy evasion in the face of other autophagy evasion mechanisms. The diversity of strain-specific interactions with the autophagy pathway amongst bacterial pathogens will also be an important question for future studies.
